# Tumor Budding beim kolorektalen Karzinom – Informationen zur klinischen Anwendung und Anleitung zur praktischen Bestimmung

**DOI:** 10.1007/s00292-021-01016-6

**Published:** 2021-11-01

**Authors:** Felix Müller, Alessandro Lugli, Heather Dawson

**Affiliations:** Institut für Pathologie, Universität Bern, Murtenstraße 31, 3008 Bern, Schweiz

**Keywords:** Lymphknotenmetastasierung, Epithelial-mesenchymale Transition, Tumorzellenverbände, Therapieplanung, Tumorgraduierung, Lymph node metastasis, Epithelial-mesenchymal transition, Tumor cell clusters, Treatment planning, Tumor grading

## Abstract

**Hintergrund:**

Bei einzelnen Patienten mit kolorektalen Karzinomen (CRC) zeigt sich ein schlechter klinischer Verlauf innerhalb desselben UICC-Stadiums (Union for International Cancer Control). Die Identifizierung von zusätzlichen Risikofaktoren ist daher notwendig, um eine optimale Therapieplanung zu erreichen.

**Fragestellung:**

In welchen Situationen kann Tumor Budding die klinische Therapieentscheidung beeinflussen und wie sollte die standardisierte Auswertung erfolgen?

**Material und Methode:**

Aktuelle Publikationen zum Thema Tumor Budding werden mit Fokus auf die praktische Anwendung und potenzielle Problemfälle in der Bestimmung des Tumor Buddings erläutert.

**Ergebnisse:**

Tumor Budding ist ein signifikanter Risikofaktor für einen schlechteren Verlauf des CRC und kann bei pT1-Karzinomen sowie Stadium-II-Karzinomen die Behandlung beeinflussen. Die Auswertung wurde durch die International Tumor Budding Consensus Conference (ITBCC) 2016 standardisiert und ist in der Praxis anwendbar. Schwierigkeiten in der Anwendung können durch die Kenntnis von potenziellen Problemfällen vermieden werden.

## Abstract (en)

**Background:**

Some patients with high-risk colorectal cancer show a worse prognosis within the same UICC stage. Therefore, the identification of additional risk factors is necessary to find the best treatment for these patients.

**Objective:**

In which settings can tumor budding help the clinical decision-making process for treatment planning and how should scoring be performed?

**Material and methods:**

Evaluation of current publications on tumor budding with an emphasis on practical grading and potential problems in the determination of tumor budding.

**Results:**

Tumor budding is a significant risk factor for worse clinical outcome of colorectal cancer and can influence clinical decision-making in pT1 and stage II colorectal cancer. A scoring method was standardized by the ITBCC 2016 and is feasible in everyday practice. Challenges in assessment can be addressed by increasing awareness of potential problem cases.

## Hinführung

Das kolorektale Karzinom (CRC) ist eine weltweit auftretende maligne neoplastische Erkrankung, welche mit einer hohen Mortalität assoziiert ist [9]. Der Standard zur Klassifizierung stellt die TNM-Klassifikation („tumour, node, metastasis“) der Union for International Cancer Control (UICC) dar. Innerhalb gleicher pT-Stadien zeigen sich unterschiedliche klinische Verläufe bei einzelnen Risikopatienten, welche beispielsweise bereits früh Lymphknotenmetastasen entwickeln [1]. Die Identifizierung von Hochrisikogruppen, welche von einer ausgedehnteren Therapie profitieren, ist daher von großer Bedeutung. Tumor Budding ist ein etablierter unabhängiger Risikofaktor für einen aggressiveren Verlauf und kann in bestimmten klinischen Szenarien ein Hilfsmittel sein, um eine Entscheidung für weitere Therapien in Form von chirurgischer Resektion oder adjuvanter Chemotherapie zu treffen [17].

## Einleitung

Tumor Budding beschreibt den Effekt von diskohäsivem Wachstum einzelner Tumorzellen bis zu Verbänden < 5 Tumorzellen im tumorassoziierten Stroma der Infiltrationszone verschiedener Karzinome (s. Infobox für die Definition eines Tumor Buds [17]). Der Effekt wurde erstmal von Imai et al. [17] in den 1950er-Jahren als „Aussprossung“ bezeichnet [15]. Tumor Budding kann im Übergangsbereich von Normalgewebe zum Karzinom als Zeichen der Infiltration (peritumorales Budding) und innerhalb eines Karzinoms (intratumorales Budding) beobachtet werden.

Im CRC konnte Tumor Budding als unabhängiger prognostischer Faktor durch zahlreiche Studien bestätigt werden [17, 22]. Es zeigte sich ebenfalls eine Korrelation mit höherem Tumorgrad, lymphovaskulärer Infiltration, Lymphknotenmetastasen und Fernmetastasen.

Als Ursache für diesen migratorischen Effekt von Tumorzellen wird die epithelial-mesenchymale Transition (EMT) postuliert, bei welcher epitheliale Zellen eine geringere Zell-Zell-Adhäsion durch Verlust von E‑Cadherin zeigen und vermehrt Marker von mesenchymalen Zellen wie Vimentin, SMA („smooth muscle actin“) oder Fibronektin exprimieren [13]. Es handelt sich hierbei um einen mehrstufigen Prozess, bei welchem die Zellen von epithelial zu mesenchymal transformieren und welcher durch die Aktivierung der Wnt/β-Catenin-Pathways bei Verlust von membranärer E‑Cadherin-Expression gekennzeichnet ist. In der aktuellen Forschung wird angenommen, dass Tumor Buds eine partielle epithelial-mesenchymale Transition (pEMT) darstellen, da etwa bei Tumor Buds des duktalen Adenokarzinoms des Pankreas keine Korrelation zwischen der membranären Reduktion von E‑Cadherin und der Expression von Vimentin besteht [13].

Die EMT kann außerhalb der Karzinogenese auch bei physiologischen Prozessen, wie z. B. der Embryogenese oder der Wundheilung, beobachtet werden. Die aberrante Expression von EMT-assoziierten Markern bei Karzinomen wird als Kennzeichen für ein erhöhtes Metastasierungsrisiko betrachtet [18].

Auch wenn Tumor Budding schon länger insbesondere beim CRC im Fokus zahlreicher Studien stand, war die Anwendung in der Praxis bis vor wenigen Jahren wegen der nicht standardisierten Auswertung limitiert. Im Rahmen der „International Tumor Budding Consensus Conference“ (ITBCC) wurde ein 2016 ein Konsens zur Auswertung des Tumor Budding mit einheitlichen Empfehlungen definiert, welche die Standardisierung der Auswertung und somit die Aufnahme in die Leitlinien ermöglichte (zusammengefasst in Tab. 1 und 2; [14, 17]). Seither wurde Tumor Budding als zusätzlicher Risikofaktor für die Progression des CRC in die Guidelines der UICC [4], den „College of American Pathologists“- (CAP-)Richtlinien [10], den „National Comprehensive Cancer Network“- (NCCN-)Richtlinien [2], der deutschen S3-Leitlinie [3] und bei den „European Society for Medical Oncology consensus“- (ESMO-)Richtlinien [5] aufgenommen.

**Table 1:** 

1	Ein Hämatoxylin/Eosin- (HE-)Schnitt mit dem höchsten Grad an Tumor Budding peritumoral wird für die Bestimmung ausgewählt (Panzytokeratinimmunhistochemie kann helfen, entsprechende Hotspot-Areale zu identifizieren)
2	Zehn Felder mit 10-facher Vergrößerung werden hinsichtlich des lokalen „Hotspots“ untersucht
3	Das Hotspot-Areal wird mittels 20-facher Vergrößerung betrachtet und die Tumor Buds ausgezählt
4	Das Ergebnis wird durch den Normalisierungsfaktor (Tab. 2) geteilt, um eine Korrektur für verschiedene Öffnungsgrößen des Okulars des Mikroskop zu erreichen
5	Das normalisierte Ergebnis ermöglicht die Einordnung in die drei Tumor-Budding-Kategorien:
	Bd1 (0–4) „low“
	Bd2 (5–9) „intermediate“
	Bd3 (10 oder mehr) „high“

**Table 2:** 

Okular Augenöffnungsdurchmesser (mm)	Betrachtete Fläche (mm^2^)	Normalisierungsfaktor zu 0,785 mm^2^
18	0,636	0,81
19	0,709	0,903
20	0,785	1
21	0,866	1,103
22	0,95	1,21
23	1,039	1,323
24	1,131	1,440
25	1,227	1,563
26	1,327	1,69

Anzahl Tumor Buds (bei 20-facher Vergrößerung)/Normalisierungsfaktor (gemäß Durchmesser Okular) = Tumor Buds (normalisiert)

Bei der ITBCC 2016 wurden folgende Aussagen definiert [17]:

1. Tumor Buds sind einzelne Tumorzellen oder Zellverbände < 5 Zellen.
2. Tumor Budding ist ein unabhängiger Prädiktor für das Auftreten von Lymphkotenmetastasen bei pT1-Karzinomen.
3. Tumor Budding ist ein unabhängiger Prädiktor für das Überleben bei Stadium-II-Karzinomen.
4. Tumor Budding sollte neben anderen klinischen und pathologischen Faktoren in einem multidisziplinären Team diskutiert werden.
5. Tumor Budding wird an Hämatoxylin/Eosin- (HE-)Schnitten bestimmt.
6. Intratumorales Tumor Budding tritt bei CRC auf und erhöht das Risiko für Lymphknotenmetastasen.
7. Tumor Budding wird in einem einzelnen „Hotspot“ (0,785 mm^2^) in der Infiltrationszone bestimmt.
8. Drei Kategorien sollten verwendet werden (Bd1 [0–4] „low“; Bd2 [5–9] „intermediate“; Bd3 [≥ 10] „high“)
9. Tumorgraduierung und Tumor Budding sind unterschiedliche morphologische Charakteristika.
10. Tumor Budding sollte in die Leitlinien/Protokolle für Tumorberichte bei CRC integriert werden.

In einer retrospektiven Studie an 771 Patienten mit CRC konnte gezeigt werden, dass Gradierung und Tumor Budding zwei statistisch unabhängige Größen sind, welche beide ein höheres pT-Stadium, Lymphangioinvasion und Fernmetastasen vorhersagen, jedoch Tumor Budding ein besserer prädiktiver Wert für das krankheitsfreie und Gesamtüberleben der Patienten ist. Gradierung und Tumor Budding sind daher nicht gleichzusetzen und können unterschiedliche biologische Prozesse darstellen [28].

Tumor Budding konnte neben dem CRC auch in verschiedenen anderen Karzinomen als relevanter Risikofaktor für einen aggressiveren Verlauf identifiziert werden, so etwa u. a. beim Plattenepithelkarzinom des Ösophagus, dem duktalen Adenokarzinom des Pankreas, dem intestinalen Typ des Magenkarzinoms, dem endometroiden Adenokarzinom des Uterus oder verschiedenen Plattenepithelkarzinomen des Kopf/Hals-Bereichs [21, 27].

## Anwendungsbereich pT1 CRC

Kolorektale Karzinome im pT1-Stadium können mittels endoskopischer Entfernung mit oder ohne onkologische Resektion (mit Lymphadenektomie) behandelt werden [3].

Grundsätzlich wird eine chirurgische Therapie bei erhöhtem Risiko für Lymphknotenmetastasen favorisiert. Das Risiko für Lymphknotenmetastasen beim pT1-CRC variiert zwischen < 1 und ca. 35 % und ist abhängig von histologisch fassbaren Risikofaktoren wie Gradierung, Gefäßinvasion, Tumorinfiltrationstiefe und Tumor Budding, wobei das Risiko mit multiplen Risikofaktoren steigt [25].

In Metaanalysen konnte gezeigt werden, dass die Odds Ratio von Tumor Budding für Lymphknotenmetastasen bei pT1-Karzinomen zwischen 6,44 und 7,74 liegt [7, 8, 11] und somit zumindest ebenso stark zum Risiko für Lymphknotenmetastasen beiträgt wie eine Lymphangioinvasion, Infiltrationstiefe und Gradierung.

In den Empfehlungen der deutschen S3-Leitlinien wird eine chirurgische Therapie bei „high risk“ pT1-Karzinomen empfohlen, welche entweder Lymphangioinvasion, hohes Grading, Tumor Budding (Bd2 oder Bd3) aufweisen [3].

## Anwendungsbereich Stadium-II-CRC

Bei Patienten mit lokal fortgeschrittenem CRC im Stadium II gemäß UICC konnte in einem systematischen Review mit insgesamt 1652 inkludierten Patienten nachgewiesen werden, dass die Gesamtüberlebenszeit bei gleichzeitigem Auftreten von „high grade“ Tumor Budding signifikant reduziert ist (Gesamtüberleben nach 5 Jahren −25 % (95 %-Konfidenzintervall (‑KI) −18 bis −33 %; *p* < 0,00001); [20]). Der prädiktive Wert von Tumor Budding für eine adjuvante Chemotherapie ist jedoch bisher nicht sicher belegt. In aktuellen Studien konnte lediglich ein bisher nicht-signifikanter Trend für ein besseres Ansprechen von Karzinomen mit hohem Tumor Budding auf adjuvante Therapie beschrieben werden [24]. Es wurde jedoch gezeigt, dass das Rezidivrisiko nach Chemotherapie bei Patienten mit „high grade“ Tumor Budding reduziert ist. Aktuell besteht keine grundsätzliche Indikation zur adjuvanten Chemotherapie bei Stadium-II-Karzinomen aufgrund des geringen Nutzens gegenüber den Nebenwirkungen (QUASAR-Studie), und diese wird nur bei einzelnen Subgruppen, wie etwa bei hoher Gradierung, pT4 oder extramuraler Veneninvasion in Erwägung gezogen [19]. Aufgrund des negativen prognostischen Werts von hohem Tumor Budding könnte es eine weitere Stütze für die klinische Entscheidung für eine adjuvante Therapie sein, jedoch ist die aktuelle Evidenz hierfür noch nicht ausreichend.

## Anwendungsbereich in präoperativen Biopsien

Neben dem etablierten peritumoralen Budding in der Infiltrationszone eines Karzinoms wurde in mehreren aktuellen Studien „intratumorales Tumor Budding“ (ITB) in Biopsien mit den pathologischen Befunden der konsekutiven Resektionspräpaten korreliert [12, 16, 29]. Beispielsweise wurde beschrieben, dass hohes Tumor Budding in Biopsien signifikant korreliert ist mit einem höheren N‑Stadium (*p* = 0,034), Fernmetastasen (*p* = 0,007), Haemangiosis carcinomatosa (*p* = 0,046), Lymphgefäßinvasionen (*p* = 0,019) und infiltrativem Wachstum (*p* < 0,001; [12]). Vor einer chirurgischen Resektion könnte aufgrund dieser Korrelation ein hohes Tumor Budding mit einer insgesamt schlechteren Prognose assoziiert sein und deshalb als Stütze für die klinische Entscheidung einer neoadjuvanten Therapie verwendet werden. Speziell beim Rektumkarzinom könnte daher eine neoadjuvante Therapie bei hohem Tumor Budding zu empfehlen sein. Bislang wurde in einer Studie niedriges Tumor Budding in präoperativen Rektumbiopsien mit einer kompletten pathologischen Tumorregression am Resektat assoziiert [16]. Hierfür sind jedoch weitere Studien nötig, um die Bedeutung von ITB zu verifizieren und Mindestanforderungen an das Biopsiematerial zu formulieren (Anzahl und Größe der Fragmente usw.).

## Ungeklärte und/oder schwierige Situationen bei der Tumor-Budding-Bestimmung

**Figure 1:**
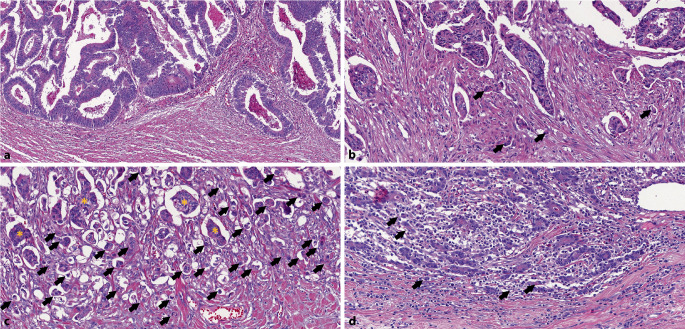

1. „Pseudobuds“: Eine Schwierigkeit für die Bestimmung der Zahl von Tumor Buds können sog. „pseudobuds“ sein. Diese entstehen durch peritumorale Inflammation, welche zur Destruktion von Drüsengewebe des CRC führt. Aus diesem Grund sollte Tumor Budding nur in Areale ausgewertet werden, in welchen keine ausgeprägte Inflammation mit Destruktion von Tumordrüsen auftritt.
2. Muzinöses und siegelringzelliges Karzinom: Tumoreinzelzellen und Siegelringzellen, welche freischwebend in Muzin liegen und auch einzeln auftreten können, sollten nicht als Tumor Buds gewertet werden. Tumor Buds sollten als solche gezählt werden, wo sie von Stroma umgeben sind. In Abwesenheit jeglicher solcher Areale sollte mit einem entsprechenden Kommentar kein Tumor Budding angegeben werden.
3. Überlagerung durch Entzündungsinfiltrate (Abb. 1): Bei dichten peri-/intratumoralen Entzündungsinfiltraten kann die Erkennung von Tumor Buds deutlich erschwert sein. In diesen Fällen ist es zulässig, zu Orientierungszwecken eine Zytokeratinfärbung durchzuführen, die eigentliche Auszählung sollte aber an der Hämatoxylin/Eosin-Färbung stattfinden [14, 17].
4. Zustand nach neoadjuvanter Therapie: Bislang gibt es keine ausreichende Studienlage, ob Tumor Budding nach neoadjuvanter Therapie eine prognostische Aussage hat. Entsprechend der aktuellen Datenlage sollte Tumor Budding in diesen Fällen noch nicht berichtet werden [14].
5. „Poorly differentiated clusters“ (PDC) und mikropapilläres Karzinom: Tumor Buds sind grundsätzlich von PDC abzugrenzen, welche definitionsgemäß aus ≥ 5 Zellen bestehen und kein glanduläres Wachstumsmuster zeigen. Auch wenn Tumor Budding separat rapportiert werden soll, ist von einem biologischen Kontinuum zwischen Tumor Buds, PDC und der mikropapillären Variante des CRC auszugehen (Abb. 1; [6]). Diese Aspekte werden allerdings noch nicht in den bestehenden Scoringsystemen widergespiegelt. Die Verwendung des Cut-off von fünf Tumorzellen zwischen Tumor Buds und PDC basiert auf der großen Anzahl veröffentlichter Studien mit dieser Definition [17]. Es besteht jedoch wenig Evidenz dafür, inwieweit dieser Cut-off geeignet ist, beide Entitäten sicher voneinander zu differenzieren.
6. Interobserver-Variabilität: Die genannten schwierigen Situationen und die Tatsache, dass Tumor Buds ausgezählt (und nicht als vorhanden bzw. nicht detektiert angegeben) werden, können dazu führen, dass zwischen verschiedenen Untersuchern unterschiedliche Werte für das Tumor Budding bestimmt werden. Die Interobserver-Variabilität lässt sich durch Ausbildung und Erfahrung reduzieren, ist aber zugleich ein potenzielles Feld für die Anwendung von automatisierten Algorithmen in der digitalen Pathologie [23].

## Ausblick nach der ITBCC 2016

Bereits bei der ITBCC 2016 war klar, dass der verfasste Konsens bei Erweiterung der Datenlage und adäquater Evidenz in der Literatur revidiert und verfeinert werden sollte. Beispielsweise wurde kürzlich eine zusätzliche Kategorie für vollständige Abwesenheit von Tumor Buds (Bd0; [26]) vorgeschlagen. In der publizierten Studie konnte gezeigt werden, dass CRC ohne Tumor Buds tendenziell niedrigere T‑Stadien und eine bessere Prognose zeigten, so dass möglicherweise die Bd0-Kategorie zusätzlich eingeführt werden könnte, um Karzinome mit günstigerem Verlauf besser charakterisieren zu können.

Ebenso sollte die mögliche automatisierte Auswertung von Tumor Budding mittels digitaler Algorithmen in künftige Richtlinien mit einbezogen werden [14, 23].

### Infobox Definition Tumor Bud [17]

Tumor Buds sind einzelne Tumorzellen oder kleine Tumorzellverbände < 5 Zellen, welche sich im Tumorzentrum (intratumorale Tumor Buds, ITB) oder peritumoral (peritumorale Tumor Buds, PTB) nachweisen lassen. Sie sind abzugrenzen von „poorly defined clusters“: Tumorzellenverbänden von ≥ 5 Zellen, welche kein glanduläres Wachstumsmuster zeigen und ebenso intra- oder peritumoral auftreten können.

## Fazit für die Praxis

- Im Zuge der Standardisierung durch die International Tumor Budding Consensus Conference (ITBCC) 2016 ist Tumor Budding ein in der Praxis einfach bestimmbarer und morphologischer Score, welcher als zusätzlicher Risikofaktor neben der TNM-Klassifikation eine Aussagekraft hinsichtlich der Prognose eines kolorektalen Karzinoms (CRC) hat. Im geeigneten klinischen Kontext (endoskopische Resektion von pT1-Karzinomen und Stadium-II-CRC) kann Tumor Budding ein Hilfsmittel für die Therapieentscheidung sein. Tumor Budding könnte zukünftig in präoperativen Biopsien ebenfalls eingesetzt werden, dies muss allerdings weiter untersucht werden.
- Für die korrekte Bestimmung des Tumor Buddings ist es notwendig, die bestehende Konsensusmethode und oben beschriebenen potenzielle Schwierigkeiten in der Bestimmung zu kennen. Weitere Studien sollten diese Aspekte und offene Fragen gezielt erforschen um in überarbeitete Richtlinien integriert zu werden.
